# Our Foes in the Air

**Published:** 1888-08

**Authors:** 


### OUR FOES IN THE AIR.

It is a wonderful fact that the air we breathe contains foes as deadly and invisible as the malignant spirits with which ancient opinion peopled it. Most epidemics are due to these unseen enemies. To be sure, it is probable that the earth and organic bodies on the earth, and not the air, generate them. They are taken up as dust, and, being little heavier than air, are long suspended in it.

They are known by the general name of microbes—microscopic forms of life. Only a few of the many classes of microbes are harmful, just as only a few of the millions of species of larger vegetable growth are poisonous.

In 1833, an epidemic fever, characterized by great depression, inflammation of the bronchial tubes and lungs, and by an incessant cough, swept over the world from east to west. Its advent was sudden, and in most cases the person was well again in less than a fortnight. It was quite fatal, however, to the aged and the weak. This was the famous influenza.

The same malady has repeated its ravages—sometimes over extensive tracts of country, sometimes only locally—many times since the Middle Ages. If it is of microbic origin,—as is probable,—what inconceivable swarms of microbes must have filled the air! Of course, no precaution could avail, except the habitual maintenance of high health and good hygienic conditions.

Malaria is due to microbes which reach their victims either from the air, by inhalation, or from drinking water which has absorbed them. Boil the water, avoid the night air, sleep in the higher rooms of the house and guard against all excesses.

Typhoid fever is caused by microbes from the discharges of a previous patient which either have found access to drinking water, or have been taken up dried into the air. Microbes flourish in all kinds of moist filth. The chief safeguard is general cleanliness, the boiling of all suspected water, and ample ventilation and sunshine.

Diphtheria, scarlet fever, whooping cough and measles are due to microbes given off from the skin or breath of the patient. They either float in the air of the room, or become attached to the furniture, walls and bedding. Avoid infected rooms and persons. Attendants should keep their stomachs in an active condition with digestible food, but should not overload them. Most microbes are digested and rendered harmless by a vigorous stomach.

The surest prophylactics—as preventives are called—are high health, cleanliness, abundance of air and sunshine, the boiling of water and milk, and the avoidance of whatever depresses the physical system—loss of sleep, protracted watching and all excesses, and cheerful courage.—*Youth's Companion.*

